# NdhV subunit regulates the activity of type-1 NAD(P)H dehydrogenase under high light conditions in cyanobacterium *Synechocystis* sp. PCC 6803

**DOI:** 10.1038/srep28361

**Published:** 2016-06-22

**Authors:** Xin Chen, Zhihui He, Min Xu, Lianwei Peng, Hualing Mi

**Affiliations:** 1 National Key Laboratory of Plant Molecular Genetics, Institute of Plant Physiology and Ecology, Shanghai Institutes for Biological Sciences, Chinese Academy of Science, 300 Fenglin Road, Shanghai 200032, China; 2 University of Chinese Academy of Sciences, Beijing 100049, China; 3 Key Laboratory of Photobiology, Institute of Botany, Chinese Academy of Sciences, Beijing 100093, China

## Abstract

The cyanobacterial NAD(P)H dehydrogenase (NDH-1) complexes play crucial roles in variety of bioenergetic reactions. However, the regulative mechanism of NDH-1 under stressed conditions is still unclear. In this study, we detected that the NDH-1 activity is partially impaired, but the accumulation of NDH-1 complexes was little affected in the *NdhV* deleted mutant (*ΔndhV*) at low light in cyanobacterium *Synechocystis* sp. PCC 6803. *ΔndhV* grew normally at low light but slowly at high light under inorganic carbon limitation conditions (low pH or low CO_2_), meanwhile the activity of CO_2_ uptake was evidently lowered than wild type even at pH 8.0. The accumulation of NdhV in thylakoids strictly relies on the presence of the hydrophilic subcomplex of NDH-1. Furthermore, NdhV was co-located with hydrophilic subunits of NDH-1 loosely associated with the NDH-1L, NDH-1MS′ and NDH-1M complexes. The level of the NdhV was significantly increased at high light and deletion of NdhV suppressed the up-regulation of NDH-1 activity, causing the lowered the photosynthetic oxygen evolution at pH 6.5 and high light. These data indicate that NdhV is an intrinsic subunit of hydrophilic subcomplex of NDH-1, required for efficient operation of cyclic electron transport around photosystem I and CO_2_ uptake at high lights.

Type-1 NAD(P)H dehydrogenase (NDH-1) complexes function in a variety of bioenergetic reactions, including respiration, cyclic electron transport around photosystem I (PSI)^1^ and CO_2_ uptake^2^ in cyanobacteria. The subunits of NDH-1 show a high homology with those in chloroplast NDH. Chloroplast NDH consists of more than 28 subunits, among those, NdhA–NdhK are plastid-encoded and others are nuclear-encoded^3^ and cyanobacterial NDH-1 consists of 17 subunits at least. Structurally, the chloroplast NDH is more complicate compared with the cyanobacterial NDH-1. According to the structural model of chloroplast NDH proposed by Ifuku *et al.*^3^, in addition to the L-shape structure consisting of subcomplex A, donor binding domain and membrane subcomplex similar with that of cyanobacterial NDH-1, the chloroplast NDH contains a subcomplex B which forms a second hydrophilic arm that extends to the stroma and attaches to the membrane subcomplex with two transmembrane proteins, PnsB4^4^ and PnsB5^5^, and the lumen subcomplex L, which consists of PnsL1-PnsL5 (photosynthetic NDH subunit of lumenal location). However, three subunits of Complex I (NuoE, NuoF, and NuoG) involved in accepting electrons from NADH in *Escherichia coli* are missing from cyanobacterial and chloroplast NDH^6^. There are six *NdhD* and three *NdhF* genes in *Synechocystis* sp. PCC 6803 (hereafter *Synechocystis* 6803) (CyanoBase, the genome database for cyanobacteria), which form different NDH-1 complexes involved in diverse physiological functions. Proteomic analysis of cyanobacterial NDH-1 complexes has revealed the presence of three complexes NDH-1L (large size), NDH-1M (medium size) and NDH-1S (small size) in *Synechosystis* 6803^7^. Further research suggested that NDH-1L functions in cyclic electron transport and respiration and NDH-1M and NDH-1S in CO_2_ uptake^8^. In contrast to the crystal structure of Complex I^9^, NDH-1 from cyanobacteria is speculated to possess an oxygenic photosynthesis-specific (OPS) domain^10^ comprised of NdhL–NdhO identified in *Synechocystis* 6803^11^,^12^. Several NDH subunits function in stabilization of NDH-1. NdhP and NdhQ were found in the purified NDH-1L complex from *T. elongatus*^13^. NdhP is involved in the respiratory and cyclic electron flow^14^ and stabilization of the NDH-1L complex^15^. NdhQ is also essential for stabilization of the large complex of NDH-1^16^. NdhS participates in the activity of cyclic electron flow around PSI in Arabidopsis^17^ or in cyanobacteria^18^, and serves as the Fd docking site domain, accepting electrons from Fd in chloroplasts^19^. Furthermore, He *et al.*^20^ have found that the NDH-1L complex interacts with Fd via the subunit NdhS in *Thermosynechococcus elongatus*.

Cyanobacteria utilize both CO_2_ and HCO_3_^−^ as carbon species^21^. The dissolved inorganic carbon is pH-dependent. With increasing pH, the ratio of HCO_3_^−^ to CO_2_ continues to rise^22^. In contrast, the water with acid pH is favorable for providing a sufficient CO_2_ supply. Cyanobacteria possess a CO_2_-concentrating mechanism (CCM) that enables to raise the concentration of inorganic carbon (Ci, HCO_3_^−^ and CO_2_) at the carboxylation site to a high level for efficient CO_2_ fixation despite the low affinity of their Rubisco for CO_2_^23^,^23^. To date, five inorganic transporters have been found including two Na^ + ^-dependent HCO_3_^−^ transporters (BicA and SbtA), one ATPase-dependent HCO_3_^−^ transporter (BCT1), and two CO_2_-uptake NDH-1 complexes in *Synechocystis* 6803 and other cyanobacterial strains^24^,^25^,^26^. As one of CO_2_ uptake systems, NDH-1MS complex, consisting of NdhD3, NdhF3 and CupA (ChyY), is inducible at limiting Ci conditions and has a higher uptake affinity for CO_2_^27^,^28^,^29^. Further research showed that the proteins encoded by *NdhF3/NdhD3/CupA/CupS* formed a small complex NDH-1S, in which CupA and a small protein CupS were identified as subunits of cyanobacterial NDH-1 by proteomic analysis^30^,^31^. NDH-1S and NDH-1M form an NDH-1MS complex which has been isolated from a *Thermosynechococcus elongatus* strain in which the C terminus of NdhL has been tagged with 6-His. This complex is easily dissociated into NDH-1M and NDH-1S complexes^31^. As a homologous gene of *cupA*, *cupB* (*chyX*) is involved in constitutive CO_2_ uptake system encoded by *NdhD4/NdhF4/CupB* which formed a small complex NDH-1S′^28^,^29^. Based on the purification of a 450 kDa complex contained both NdhH and CupB protein, it has been suggested that the complex is NDH-1MS′ located in the thylakoid membranes^32^.

Previous studies have shown that the cyclic electron flow around PS I mediated by NDH functions in protection against stressed conditions. Ogawa^2^ has found that deletion of *NdhB* gene resulted in the unable survival phenotype of *Synechocystis* 6803 under low CO_2_ (air) condition, suggesting that the NDH-1 functions in inorganic carbon transport. The activity of cyclic electron flow around PSI mediated by NDH-1 has been found to be enhanced under stressed conditions, including low CO_2_^33^, strong light^34^. However, how NDH-1 participates in the regulation of photosynthesis is still unclear.

Recently, NdhV is reported to function in stabilization of chloroplast NDH-like complex in Arabidopsis^35^ and heat tolerance in *Synechocystis* 6803^36^. However, the mechanism of NdhV in response to stressed conditions is not clear. Here we report that cyanobacterial NdhV localizes in the hydrophilic subcomplex of NDH-1MS′, NDH-1L and NDH-1M. Further analysis showed that NdhV functions in regulation of NDH-1 activity for efficient operation of cyclic electron flow around PS I and CO_2_ uptake in *Synechocystis* 6803 in response to high lights.

## Results

### Deletion of *NdhV* partially arrests NDH-1 activity

**Figure 1:**
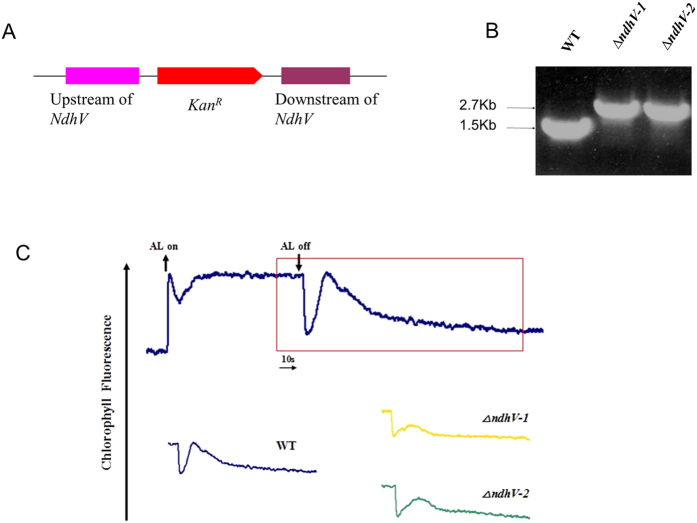
*NdhV* gene deletion and its effect on NDH-1 activity. (**A**) Construction of plasmid to generate *NdhV* deleted mutant (*ΔndhV*). Schematic representation of the *ΔndhV* mutant locus. A kanamycin resistance cassette about 1.2Kb was inserted into the *NdhV* gene. (**B**) PCR segregation analysis of the *ΔndhV* mutant using the *ndhV*-up-F and *ndhV*-Dn-R primers (Table S1). (**C**) Monitoring of NDH-1 activity using chlorophyll fluorescence analysis. The top curve shows a typical trace of chlorophyll fluorescence in the WT of *Synechocystis* 6803. The cells (OD_730_ around 0.4) supplemented with 10 mM NaHCO_3_ were used for the measurement. After the sample was exposed to the actinic light (AL, 100 μmol photons m^−2^ s^−1^) for 90 s, AL was turned off, and the transient increase in chlorophyll fluorescence level was recorded, which was used to ascribe NDH-1 activity. The inset shows magnified traces from the box area.

To reveal the function of *NdhV* in cyanobacteria, we inactivated the cyanobacterial NdhV protein by inserting a kanamycin resistance (*kan*^*R*^) cassette into its coding region (Fig. 1A). The PCR analysis of the *NdhV* locus using the *ndhV*-up-F and *ndhV*-Dn-R primers showed a band of 1.5 Kbp in the WT and 2.7 Kbp in the *ΔndhV* mutant which contained an extra *Kanamycin* resistance cassette of about 1.2 Kbp; it confirmed the complete segregation of the *ΔndhV* allele in this mutant (Fig. 1B).

Post-illumination increase in Chl fluorescence was explained as the reduction of plastoquinone (PQ) by the electrons from photoreductants accumulated in the stroma or cytosol during illumination, which reflects cyclic electron transport around PSI mediated by NDH-1^37^,^38^ in cyanobacteria and by chloroplast NDH in higher plants^39^. By comparison with WT, the post-illumination increase in Chl fluorescence was partly impaired in two lines of *ndhV* deleted mutants *ΔndhV-1* and *ΔndhV-2* (Fig. 1C), suggesting that NdhV contributes to the NDH-1 activity.

### Growth of *ΔndhV* was suppressed under high light and low pH conditions

**Figure 2:**
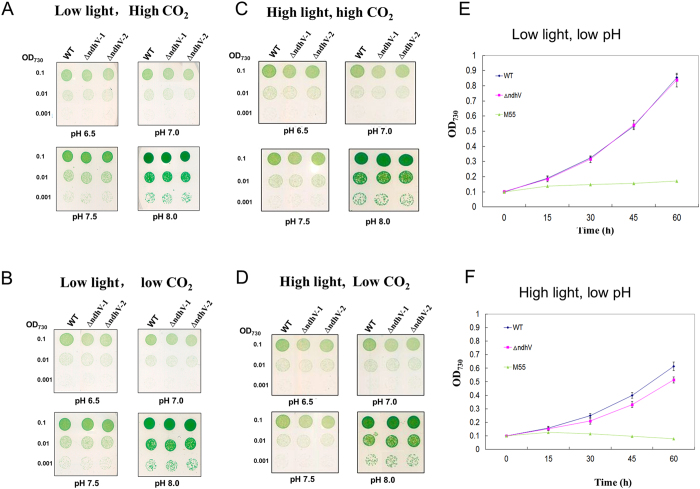
The growth phenotype of WT and *ΔndhV* strains. (**A–D**) Five microliters of the cell suspensions with the OD_730_ nm values of 0.1, 0.01, and 0.001 were spotted on agar plates containing BG11 buffer at different pHs and grown at a low light (40 μmol photons m^−2^ s^−1^), high CO_2_ (3% v/v) (**A**); low light, low CO_2_ (0.04% v/v) (**B**); high light (300 μmol photons m^−2^ s^−1^) and high CO_2_ (**C**); ligh light and low CO_2_ (**D**) for five days. (**E**,**F**) Cell density of WT, *ΔndhV* , and M55 strains were measured at different times after grown at low light (40 μmol photons m^−2^ s^−1^) and low pH (pH 6.5) (**E**), or high light (300 μmol photons m^−2^ s^−1^) and low pH (pH6.5) (**F**), 2% CO_2_ (v/v in air). Values are means ± SD (*n* = 3). Values are means ± SD (*n* = 3).

In addition to cyclic electron flow around PSI, NDH-1 is also involved in CO_2_ uptake^2^. To examine whether NdhV participates in CO_2_ uptake, we compared the growth phenotype between WT and *ΔndhV* under various conditions, low (40 μmol photons m^−2^ s^−1^) or high light (300 μmol photons m^−2^ s^−1^), low (below pH 7.5), or high pH (above pH 7.5), high (2%) or low CO_2_ (0.04%). With the pH increasing to higher than 7.0, the concentration of HCO_3_^−^ is predominant but that of CO_2_ becomes minor, while at the low pH below 6.5 condition, the concentration of HCO_3_^−^ becomes minor and that of CO_2_ is predominant. The growth phenotype was almost the same under pH 8.0 either at low light or high light (Fig. 2A–D). The growth of *ΔndhV* was slightly slow at low pH, low light, low CO_2_ (Fig. 2B) or high light, high CO_2_ (Fig. 2C) and more evidently under high light, low CO_2_ (Fig. 2D). To confirm the difference, we further compared the growth rate in the liquid culture conditions bubbling with 2% CO_2_ at pH 6.5 at low light or high light. The growth rate was identical between *ΔndhV* and wild type under low light condition in liquid (Fig. 2E), consistent with the results on agar (Fig. 2A). However, the growth of *ΔndhV* was slower than WT under the high light condition (Fig. 2F), suggesting that NdhV plays an important role under the stressed conditions. In contract, the *ndhB* defective mutant (M55) in which both the PSI-cyclic electron flow and CO_2_ uptake were inactivated, could hardly grow up at pH 6.5 under either low light or high light conditions (Fig. 2E,F), similar to the observation by Ogawa (1991).

### Suppression of the activity of CO_2_ uptake in *ΔndhV*

**Figure 3:**
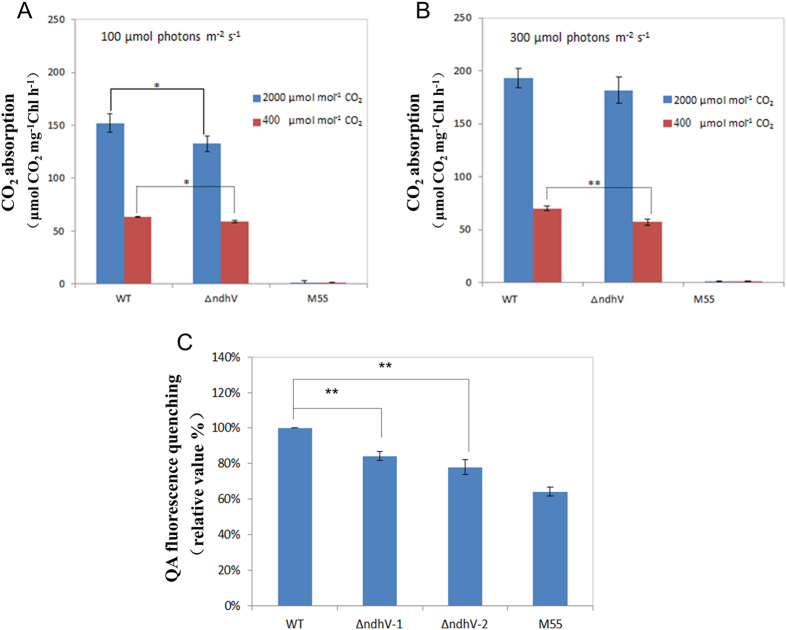
Comparison of the CO_2_ uptake and the proton gradient across thylakoid membranes between WT and *ΔndhV* strains. (**A,B**) The rate of CO_2_ uptake in WT, *ΔndhV* and M55 strains at 100 μmol photons m^−2 ^s^−1^ (**A**) or at 300 μmol photons m^−2 ^s^−1^ (**B**). The cells of WT and mutant strains were harvested at mid-logarithmic phase (OD_730_ ≈ 0.5) and chlorophyll *a* concentration was adjusted to 400 μg ml^−1^. 30 μl of the cell suspensions were placed on the BG-11 agar plate. The activity of CO_2_ uptake was measured at 30 °C. The CO_2_ concentration was controlled at 400 or 2000 μmol mol^−1^. Values are means ± SE of three independent measurements. Asterisk indicates significant differences (t-test, *P < 0.05 and **P < 0.01). (**C**) Analysis of proton gradient across thylakoid membranes using QA (quinacrine) fluorescence quenching in WT, *ΔndhV* and M55 strains. Intact cells of WT and mutant strains were harvested at mid-logarithmic phase (OD_730_≈0.5) and then suspended at a final chlorophyll a concentration of 150 μg ml^−1^ in a reaction medium contained 5 mM Tris/MES (pH 8.0), 0.3 M mannitol, 2 mM DTT, 5 mM D-Glucose, 1.5 mM ATP, 5 μM quinacridine. The quenching of QA fluorescence was induced by adding the cells sample to 2 ml reaction mixture after the background fluorescence reached steady state about 2 min after started the measurement. The QA fluorescence quenching of WT is 5.02%. Values are means ± SE of three independent measurements. Asterisk indicates significant differences (t-test, *P < 0.05 and **P < 0.01).

To confirm whether the CO_2_ uptake activity was affected by deletion of NdhV, we compared the rate of CO_2_ uptake in WT, *ΔndhV*, and M55 in which only NDH-1S is active in the CO_2_ acquisitive systems, using a portable open-flow gas exchange system under high lights and high or low CO_2_ conditions. Cell suspensions were placed on the BG-11 agar plate to measure the CO_2_ uptake rate. The rate of CO_2_ uptake was lowered by about 10% in *ΔndhV* than that in WT at 100 μmol photons m^−2^ s^−1^ either under high CO_2_ or low CO_2_ conditions (Fig. 3A), and at 300 μmol photons m^−2^ s^−1^ under low CO_2_, but not under high CO_2_ condition (Fig. 3B). In contrast, the rate of CO_2_ uptake was almost completely suppressed in M55 (Fig. 3A,B) suggesting the loss of function of both NDH-1MS and NDH-1MS′ in the mutant. These results demonstrated that deletion of *NdhV* suppresses the CO_2_ uptake activity under high lights and CO_2_ limitation conditions.

### The proton gradient across thylakoid membranes was lowered in *ΔndhV*

It has been suggested that cyanobacterial NDH-1 provides ATP for CO_2_ uptake^2^. To investigate whether NdhV is involved in this process, the proton gradient across thylakoid membranes, which drives ATPase to synthesis ATP was compared between WT and *ΔndhV*. Quinacrine (QA) fluorescence quenching can be used for the determination of *Δ*pH across the thylakoid membrane for intact cells of *Synechocystis* 6803^40^. The QA fluorescence quenching was decreased by about 20% in *ΔndhV* mutants and 40% in M55 compared with those in WT (Fig. 3C), suggesting that NdhV is involved in regulation of proton gradient across thylakoid membrane.

### NdhV localizes to the thylakoid membrane and its accumulation relies on the electron donor domain of NDH-1M of NDH-1MS′, NDH-1L

**Figure 4:**
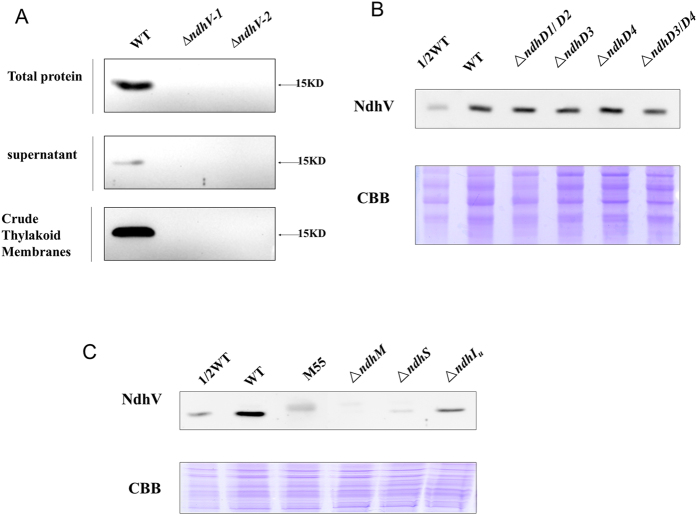
The location of NdhV in WT strain and the effects of mutation of Ndh subunits on NdhV. (**A**) Immunodetection of NdhV in the total proteins, supernatant and thylakoid membranes of WT and *ΔndhV* strains. Total Proteins: The material obtained after broken by glass beads; supernatant, Crude Thylakoid Membranes: supernatant and precipitation after centrifugation of total proteins at 20, 000 × *g* for 30 min at 4 °C, respectively. (**B**) Immunodetection of NdhV in thylakoid membranes from WT (including indicated serial dilutions), *ΔndhD1/D2*, *ΔndhD3*, *ΔndhD4* and *ΔndhD3/D4* mutants. Immunoblotting was performed using antibodies against NdhV. Each Lane was loaded with 25 μg total proteins. In the lower panel, a piece of replicated gel stained with Coomassie Brilliant Blue (CBB) was used as a loading control. (**C**) Immunodetection of NdhV in thylakoid membranes from WT (including indicated serial dilutions), M55, *ΔndhM*, *ΔndhS*, *ΔndhI*_*U*_(partly deletion of NdhI) mutants. Immunoblotting was performed using antibodies against NdhV. Each Lane was loaded with 25 μg total proteins. In the lower panel, a piece of replicated gel stained with Coomassie Brilliant Blue (CBB) was used as a loading control.

The previous study demonstrated that Arabidopsis NdhV is mainly localized to the thylakoid membrane^35^,^36^. As a homologous protein, the localization of the cyanobacterial NdhV was investigated with a polyclonal antibody against the recombinant cyanobacterial NdhV. Western blotting analysis detected a band with a molecular mass of about 15 kDa (the theoretical molecular mass of mature NdhV is ~18 kDa) in the thylakoid and also in the stromal fractions of WT, but absent in those of *ΔndhV* (Fig. 4A), confirming that the cyanobacterial NdhV is mainly localized to the thylakoid membrane.

In cyanobacteria, a total of 4 NdhD isoforms are present and they are located in different NDH-1 complexes. Given that NDH-1L, NDH-1MS, NDH-1MS′ were respectively defective in *ΔndhD1/D2, ΔndhD3* and *ΔndhD4* mutant in *Synechocystis* 6803^30^, suggesting that NDH-1M is stable in those *NdhD* deleted mutants, we checked the amount of NdhV in different NdhD deleted mutants. As shown in Fig. 4B, there was no much difference in the amount of NdhV among WT and the various *NdhD* deleted mutants, suggesting that NdhV is present in NDH-1M complex.

To further confirm the association of NdhV with NDH-1M, the accumulation of NdhV in the mutants defective in different Ndh subunits was investigated by immunoblot. Figure 4C shows that NdhV was detected neither in NdhB defective mutant M55, in which both the NDH-1L and NDH-1M were disassembled^30^ nor in *NdhM* deleted mutant *ΔndhM* in which all the NDH-1 complexes were disassembled^41^. The results indicate that the accumulation of NdhV relies on NDH-1M and the complexes containing NDH-1M, including NDH-1L, NDH-1MS and NDH-1MS′.

We further checked the accumulation of cyanobacterial NdhV in *NdhS* deleted mutant of *Synechocystis* 6803 (*ΔndhS*). NdhV was hardly detected in *ΔndhS* (Fig. 4C), consistent with the result obtained in Arabidopsis *ndhS/crr31* mutant (Fan *et al.*)^35^ or in *Synechocystis* 6803 (Gao *et al.*)^36^, suggesting that the stability of NdhV is dependent on the electron donor domain of NDH-1 in cyanobacteria. Furthermore, we checked the accumulation of NdhV in the mutant defective in the hydrophilic subunits of NDH-1. The amount of NdhV was reduced significantly in *NdhI* partly deleted mutant (*ΔndhI*_*U*_) (Fig. 4C). The result indicates that the accumulation of NdhV is dependent on the presence of the hydrophilic subcomplex of NDH-1related to its electron donor domain.

### NdhV is loosely bound in NDH-1 complexes and its absence hardly affects the accumulation of other Ndh subunits and assembly of NDH-1 complexes under normal growth conditions

**Figure 5:**
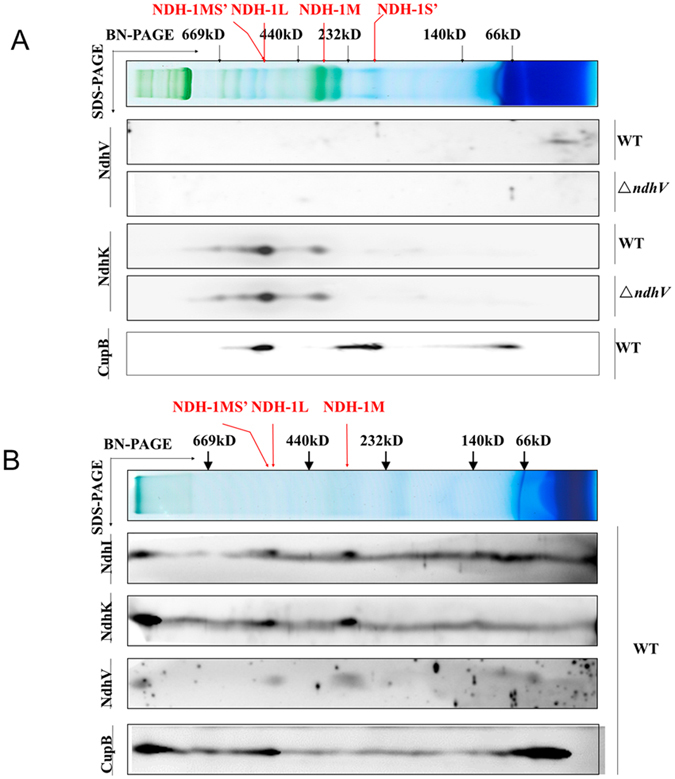
The location of NdhV in NDH-1 complexes in WT. (**A**) Thylakoid membrane proteins from WT and *ΔndhV* strains were separated by the BN-PAGE and further subjected to a 2D/SDS-PAGE. The proteins were immunodetected with antibodies against NdhK and NdhV. The positions of molecular mass markers in the BN-gel are indicated. (**B**) Thylakoid membrane proteins from WT strain were crosslinking by DSP, then separated by BN-PAGE and further subjected to 2D/SDS-PAGE. The proteins were immunodetected with antibodies against NdhI, NdhK, NdhV and CupB. The positions of molecular mass markers in the BN-gel are indicated.

To investigate whether the accumulation of the NDH-1 complexes is affected in *ΔndhV,* thylakoid protein complexes isolated from *ΔndhV* and WT were separated by a 5–13% gradient blue-native PAGE (BN-PAGE) followed by 2D SDS-PAGE for the immunoblotting analysis. The results show that in the wild type, NdhV presents as a free form and the bands corresponding to NDH-1L and NDH-1M complexes were detected using the antibody of NdhK, and the bands corresponding to NDH-1MS′ and NDH-1S′ complex could also be detected using the antibody of the key component CupB (Fig. 5A). However, in *ΔndhV,* these bands were almost the same as those in wild type, indicating that deletion of NdhV hardly affected the assembly of NDH-1 complexes, including NDH-1L, NDH-1MS′ and NDH-1M (Fig. 5A), in accordance with the results in Arabidopsis that the NdhV is a protein easily dissociated from NDH-1 complexes during sample preparation and BN-gel electrophoresis. To overcome this limitation, we performed the chemical crosslinking experiments using the thylakoids isolated from WT. The result shows that NdhV co-migrates with the NdhI and NdhK subunits in the NDH-1M, NDH-1L, NDH-1MS′ and a supercomplex around 1000 kDa, probably due to the aggregation of the NDH-1 (Fig. 5B). These results also confirm that NdhV is a subunit of cyanobacterial NDH-1 but easily dissociated from NDH-1 complexes. To know whether NdhV affects the accumulation of other subunits of NDH-1, we checked the amount of several subunits in different parts of cells from *ΔndhV*. The results show that there were no obvious difference of the amount of NdhH, I, K, M either in total proteins, or in the supernatant or in the thylakoid membrane between the WT and *ΔndhV* cultured at pH 6.5 (Fig. 1SA) or at pH 8.0 (Fig. 1SB).

### The up-regulation of NDH-1 activity at high lights was suppressed in *ΔndhV*

**Figure 6:**
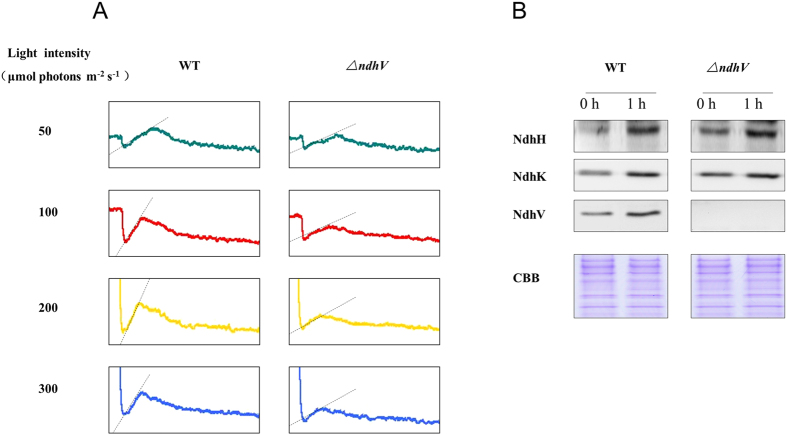
Monitoring of NDH-1 activity of WT and *ΔndhV* strains under different light intensities. (**A**) Monitoring of NDH-1 activity of WT and *ΔndhV* strains in different light intensities using chlorophyll fluorescence analysis. OD_730_ of the cells was about 0.3. The cells were exposed to the different actinic light (shown in the figure) for 90 s. Then the actinic light was turned off, and the transient increase in chlorophyll fluorescence level was ascribed to NDH activity. (**B**) Immunodetection of NdhH, K, V in total proteins of WT and *∆ndhV* strains before and after treatment with high light. The cell was cultured to mid-logarithmic phase under normal light (0 h), then the cell cultures were transferred to high light (~200 μmol photons m^−2^ s^−1^) for 1 hour (1 h). Immunoblotting was performed using antibodies against NdhH, NdhK and NdhV. Each lane was loaded with 25 μg proteins. In the lower panel, a piece of replicated gel stained with Coomassie Brilliant Blue (CBB) was used as a loading control.

Previous studies have indicated that NDH-1 activity was up-regulated by high light^34^. To test whether NdhV is required for up-regulation of NDH activity under high light conditions, we compared the NDH-1 activity under different light intensities between WT and *ΔndhV*. Figure 6A shows that the NDH-1 activity, reflected by the transient increase in Chl fluorescence after termination of actinic light, increased with the increase of the intensity of actinic light in WT, but the up-regulation of NDH-1 activity in response to high lights was significantly suppressed in *ΔndhV*. The results indicate that the up-regulation of NDH-1 activity is almost suppressed when NdhV is absent. Furthermore, we compared the changes of the amount of Ndh subunits after adaptation to the high light between WT and *ΔndhV.* As shown in Fig. 6B, after the growth light was shifted to the high light (200 μmol photons m^−2^ s^−1^) for 1 h, the amounts of NdhH and NdhK were evidently increased by about 40% and 35%, respectively, both in WT and *ΔndhV.* Meanwhile, the amount of NdhV was approximately 2-fould higher at the high light in WT. The results suggest that the up-regulation of NdhV is required for regulation of NDH activity in response to high lights in cyanobacteria.

### The capacity of photosynthesis was suppressed at the high light and low pH in *ΔndhV*

**Figure 7:**
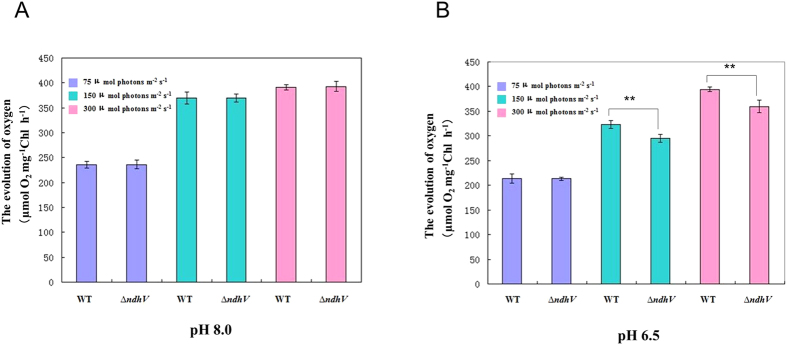
The evolution of oxygen in WT and *ΔndhV* strains at different light intensities and different pHs. (**A,B**). The evolution of oxygen in WT and *ΔndhV* strains under different light intensities at pH 8.0 (**A**) and 7.0 (**B**). WT and *ΔndhV* strains were grown at 30 °C in BG-11 medium buffered with 5 mM Tris-HCl at pH 8.0 and 7.0, respectively, in 2% CO_2_ (v/v in air) at 50 μmol photons m^−2^ s^−1^. Then the cells at logarithmic phase (OD_730_ ≈ 0.5) were used to measure the evolution of oxygen in the presence of 200 μM NaHCO_3_ at the light intensities of 70, 150 and 300 μmol photons m^−2^ s^−1^, respectively. Values are means ± SE of five independent measurements. Asterisk indicates significant differences (t-test, *P < 0.05 and **P < 0.01).

To check whether NdhV functions in protecting the cyanobacterium against the high light stress, we compared the capacity of photosynthesis between WT and *ΔndhV* in response to the high light, as reflected by photosynthetic O_2_ evolution at higher pH (8.0) where the HCO_3_^−^ is predominant and lower pH (6.5) where CO_2_ is predominant. With the increase in the light intensity, the rate of photosynthetic O_2_ evolution increased, but there was not much difference between WT and *ΔndhV* at pH 8.0 (Fig. 7A). However, the rate of photosynthetic O_2_ evolution in *ΔndhV* was approximately 10% lower than that in WT at pH 6.5 at higher light intensities (Fig. 7B). The results suggest that NdhV is also required for photosynthetic CO_2_ assimilation under the high light and low pH stressed conditions.

## Discussion

It has been shown that mutation of *NdhV* caused completely the loss of the NDH activity in Arabidopsis^35^ but only partly in cyanobacterium *Synechocystis* 6803 (Gao *et al.*)^16^, which is confirmed in our work (Fig. 1). The only partial suppression of the NDH-1 activity (Fig. 1C) by deletion of the cyanobacterial *NdhV* is probably attributed to the compensation of the activity from different types of NDH-1 in cyanobacteria^7^. On the other hand, similar with the chloroplast NdhV, the cyanobacterial NdhV is also demonstrated to localize in the fragile part of NDH-1 and may interact with the electron donor domain of NDH-1 by the observation of the severe suppression of the accumulation of NdhV in *ΔndhS* (Fig. 4C). In addition, the accumulation of NdhV was almost lost in the thylakoid membranes in M55 and in *ΔNdhM* (Fig. 4C) in which the hydrophilic subcomplex of NDH-1 complexes are degraded (He *et al.*)^20^, but it was not affected either in *NdhD3/D4* defective mutant (Fig. 4B) in which only NDH-1L and NDH-1M exist, or in *NdhD1/D2* defective mutant (Fig. 4B) in which NDH-1M, NDH-1MS′ are still present. Moreover, NdhV co-localized with Ndh subunits of NDH-1MS′, NDH-1L and NDH-1M complexes (Fig. 5A). Considering the cells were cultured under high CO_2_ culture condition, NDH-1MS would not be visible. Those results let us conclude that NdhV interacts with the electron donor domain of these complexes which contains NDH-1M as a skeleton^30^. Although defect in NdhS and other hydrophilic subunits affected the accumulation of NdhV (Fig. 4C), but deletion of *NdhV* didn’t affect the accumulation of relevant NDH-1 subunits nor the assembly of NDH-1 complexes (Fig. 1S), implying that NdhV is a peripheral subunit functioning in regulation of NDH-1.

The growth phenotype of both wild type and *∆ndhV* cultured in liquid medium bubbling with 2% CO_2_ at pH 6.5 and low light was almost the same (Fig. 2E), which was consistent with the previous observation (Gao *et al.*)^16^ who therefore concluded that NdhV didn’t play any role in CO_2_ uptake. However, when *∆ndhV* was cultured in agar plate in 2% CO_2_ at pH 6.5 and low light, the growth was slightly suppressed (Fig. 2B), suggesting that the bubbling may increase the diffusion of HCO_3_^−^ into the carboxysome even if it was not predominant, but the ratio of CO_2_ to HCO_3_^−^ to is still about 0.7 at pH6.5. In addition, the growth of *∆ndhV* was evidently suppressed at high light when HCO_3_^−^ or CO_2_ were limited at low pH or low CO_2_ (Fig. 2C,D,F) but not significantly at high light when the inorganic carbon is saturated at high pH or high CO_2_ (Fig. 2C,D). The results suggest that NdhV is required for the efficient CO_2_ uptake at high light especially under the conditions when the inorganic carbon is limited, which is further supported by the observation that the CO_2_ uptake activity was evidently suppressed at the high light of 300 μmol photons m^−2^ s^−1^ under low CO_2_ but not under high CO_2_ condition (Fig. 2C) and also by the suppression of photosynthetic O_2_ evolution at the high light at low pH (Fig. 7B) but not at high pH (Fig. 7A). Defect in the cyanobacterial NdhV caused partially (about 10%) suppression of the rate of CO_2_ uptake (Fig. 3A), it might be attributed to the partly contribution of NdhV to the cyclic electron or respiratory electron transports which might function in providing transthylakoid membrane proton gradient (Fig. 3C) for ATP synthesis or alkaline pocket (Kaplan and Reinhold, 1999).

The previous study showed that both the cyclic electron flow around PSI and respiratory O_2_ uptake mediated by NDH-1 was enhanced at high light (100 μmol photons m^−2^ s^−1^), accordingly the photosynthetic O_2_ evolution was also up-regulated^34^, suggesting that the up-regulation of NDH-1 is required for high activity of photosynthesis. The up-regulation of the NDH-1 activity induced by the high lights was suppressed when *NdhV* is deleted (Fig. 6A), suggesting that the cyanobacterial NdhV is involved in the regulation of NDH-1 activity in response to the high lights. The significant up-regulation of NdhV by the treatment of the high light in the wild type (Fig. 6B) implies its important role in the regulation of photosynthesis, as one of components related with the electron donor domain of NDH-1. The evident suppression of the rate of CO_2_ uptake at the high light (100 μmol photons m^−2^ s^−1^) either at high CO_2_ or low CO_2_ (Fig. 3A) further supports this mention. In addition, the suppression of the photosynthetic oxygen evolution (Fig. 7B) and the growth (Fig. 2D,F) at the high light when CO_2_ was limited in the NdhV deleted mutant might be attributed to the loss of the up-regulation for NDH-1 activity under the stressed conditions (Fig. 6A). It has been indicated that cyclic electron flow around PSI plays an important role in providing ATP for carbon assimilation^42^,^43^. Actually, the enhancement of cyclic electron flow around PS I by treatment with low concentration of NaHSO_3_ enables cyanobacteria to generate sufficient proton gradient across the thylakoid membrane, thereby increased the biomass of *Synechocystis* PCC 6803^44^. Therefore, in NdhV deleted mutant, the partial suppression of NDH-1 activity (Fig. 1C) would lower the building-up of proton gradient across thylakoid membranes (Fig. 3C), resulting in the decrease in the activity of CO_2_ uptake (Fig. 3) and suppressed growth, especially when HCO_3_^−^ and CO_2_ were limited under high light conditions (Fig. 2D).

In conclusion, our work demonstrate that the cyanobacterial NdhV subunit locates at the hydrophilic parts of NDH-1M and plays an essential role in regulation of NDH-1 activity for efficient operation of cyclic electron flow around PS I and CO_2_ uptake especially at high light.

## Materials and Methods

### Cell culture conditions

The *Synechocystis* 6803 wild type and mutant cells were cultured in BG-11 medium^45^ buffered with Tris-HCl (5 mM, pH 8.0) at 30 °C under 50 μmol photons m^−2^ s^−1^ . The cells were bubbled with 2% v/v CO_2_ in air. The BG-11 solid medium used was BG-11 supplemented with 1.5% agar, Tris-HCl (10 mM, pH 8.0), and 0.3% Na_2_S_2_O_3_. The illumination was provided by fluorescence lamps at 50 μmol photons m^−2^ s^−1^. According to culturing requirements of wild type and mutant cells, the BG-11 medium was buffered with 10 mM TES-KOH at several other pH conditions.

### Construction of *ΔndhV* mutant

The upstream and downstream regions of *sll0272* (*NdhV*) and a DNA fragment encoding a kanamycin resistance (Kan^R^) cassette were firstly amplified by PCR using specific oligonucleotide primers (Table S1). Then the PCR products of upstream of *sll0272* and *Kanamycin resistance* (*Kan*^*R*^) cassette were used as templates to obtain the second PCR products(SP1), and the PCR products of downstream of *sll0272* and *Kan*^*R*^ cassette were used as templates to obtain the second PCR products(SP2). The third round PCR product containing the upstream and downstream regions inserted by a *Kan*^*R*^ cassette were amplified by PCR using SP1 and SP2 as templates. Then the final products were ligated to the pMD19T (Fig. 1A), to make the construct for transforming the WT cells of *Synechocystis* 6803 as described by Williams and Szalay^46^. The transformants were spread on agar plates containing BG-11 medium and Kanamycin (10 μg.ml^−1^) buffered at pH 8.0, the BG-11 solid medium plates were incubated in 2% v/v CO_2_ in air. The mutated *NdhV* in the transformants was segregated to homogeneity as determined by PCR amplification and immunoblotting.

### The measurement of chlorophyll fluorescence

The transient increase in chlorophyll fluorescence after actinic light had been turned off was monitored by means of using a PAM Chl fluorometer (Walz, Effeltrich, Germany), emitter-detector-cuvette assembly (ED-101US) and unit 101ED as previously described^37^,^38^.

### Oxygen exchange

The evolution of oxygen under different light intensities were determined in cultural BG-11 medium that contained the mid-logarithmic cell of *Synechocystis* 6803 at about 2.5 μg chlorophyll ml^−1^ with a Clark-type oxygen electron. The whole monitoring process was performed at 30 °C.

### CO_2_ uptake measurement

CO_2_ uptake was measured with a portable open-flow gas exchange system Li-6400 (LI-COR Biosciences). Air temperature of the leaf chamber was maintained at 30 °C, the photosynthetically active radiation (PAR) was 100 or 300 μmol photons m^−2^s^−1^ and the flow rate of the air in the measuring chamber was 50 μmol s^−1^. CO_2_ concentration was controlled at 2% (v/v in air) or 0.04%. The cells of WT and *ΔndhV* strains grown at 30 °C in BG-11 medium at 2% CO_2_ were collected at mid-logarithmic stage by centrifugation and re-suspended in fresh BG-11 medium. They were concentrated into 100 of OD_730_, and 30 μl of each cell suspension was placed on the agar plate as a spot incubated in the growth chamber before CO_2_ uptake measurement. Each cell spot on agar was cut out into a square of 1 cm × 1 cm and put on a cover glass to measure the CO_2_ uptake rate. Same size of agar was used as control. Measurements were repeated three times and the averages were recorded.

### Isolation of crude thylakoid membranes

*Synechocystis* 6803 thylakoid membranes were isolated as described by Gombos *et al.*^47^ with some modifications as follows. The cell cultures (1L) were centrifuged at the logarithmic phase and then resuspended in 20 ml disruption buffer [10 mM HEPES-NaOH, 5 mM sodium phosphate, pH 7.5, 10 mM MgCl_2_, 10 mM NaCl, and 20% v/v glycerol)], broken by vortexing with glass beads (150–212 μm) for three times at 70 Hz for 30 s with 30 s interval cooling using a Tissuelyser-48 system (Shanghi Jingxin). And then the lysis was centrifuged at 5000 g for 5 min at 4 °C to remove glass beads and unbroken cells. The crude thylakoid membranes and supernatant were obtained by centrifugation of the homogenate at 20 000 g for 30 min at 4 °C from the precipitation and supernatant, respectively. The membranes were washed with buffer B [330 mM sorbitol, 50 mM Bis-Tris-HCl, pH 7.0, 0.5 mM PMSF (Sigma, MO)] and re-suspended in buffer C [25 mM BisTris-HCl, pH 7.0, 10 mM MgCl_2_, 20% v/v glycerol, 0.1 units RNase-free DNase RQ1 (Promega, Madison, WI), 0.5 mM PMSF].

### Cross-linking of the thylakoid membranes

The cross-linking assay was performed as described previously^48^ . Thylakoid membranes were resuspended in 1 ml of 20 mM Hepes-KOH, pH 8.0, 5 mM MgCl_2_ (0.5 mg chlorophyllml^−1^) and incubated with 2.5 mM DSP (Dithiobis[succinimidyl propionate]) for 30 min at room temperature in the dark. After cross-linking, reactions were quenched with addition of 60 mM Tris-HCl, pH 7.5 for 15 min. Thylakoids were pelleted after centrifuged again at 20 000 g for 30 min at 4 °C and solubilized at 0.5 mg of chlorophyll ml^−1^ in a buffer containing 20 mM Hepes-KOH, pH 8.0, 200 mM NaCl, 1.2% Triton X-100, 1 mM PMSF for 30 min on ice. Insoluble thylakoids were removed by centrifugation at 22 000 g for 10 min at 4 °C and the supernatant was transferred to new tubes for further analysis.

### Electrophoresis and immunoblotting

BN-PAGE of *Synechocystis* 6803 membranes was performed as described previously^48^ with slight modifications. The chlorophyll a concentration of the membranes solubilized in buffer C was diluted at 0.5 mg ml^−1^, then the 1/20 vloume of 20% *n*-dodecyl-β-D-maltoside (DM) was added. After incubation on ice for 20 min and centrifugation at 20 000× *g* for another 15 min, the supernatants were supplemented with 1/10 volume of BN sample buffer (5% Serva Blue G, 100 mM BisTris-HCl, pH 7.0, 30% w/v sucrose, 500 mM ε-amino-*n*-caproic acid and 10 mM EDTA). Solubilized membranes were then applied to a 1.0 mm thick 5–13% acrylamide gradient gel. Samples of 5 μg chlorophyll *a* were loaded on the gel. Electrophoresis was performed at 4 °C by increasing the voltage gradually from 50–200 V during the 5 h run. For electrophoresis in the second dimension, the lanes of the BN gel were cut out and incubated in 1× SDS sample loading buffer containing 2% β-mercaptoethanol for 30 min. SDS-PAGE of the lanes of the BN gel was performed on a 15% polyacrylamide gel as described previously^49^.

For immunoblotting, the proteins were electrotransferred to a polyvinylidene difluoride (PVDF) membrane (Immobilon-P; Millipore) and detected using protein-specific antibodies with the ECL assay kit (BioRad) according to the manufacturer’s protocol. Antibody against the NdhV protein of *Synechocystis* 6803 was raised in our laboratory. To amplify the *NdhV* gene, primer sequences were listed in Table S1. The PCR product was ligated into vector pET51b (Novagen). The plasmid was used to transform *E.coli* strain BL21 (DE3) pLysS for expression. Polyclonal antibody was raised in a rabbit from purified recombinant protein. The antibodies against CupB, NdhH, NdhI, NdhK, and NdhM were previously raised in our laboratory.

### Quinacrine (QA) fluorescence quenching

Fluorescence of Quinacrine (QA) at 503 nm using the PAM chlorophyll fluorometer (Maxi-version, Walz, Effeltrich, Germany) attached with a US-370 emitter with an emission peak at 375 nm and a PM-101/D detector as described previously^50^. Cells were harvested at logarithmic phase and suspended in reaction mixture contained 5 mM Tris/MES (pH 8.0), 0.3 M mannitol, 2 mM DTT, 5 mM D-Glucose, 1.5 μM ATP, 2.5 μM QA with a final chlorophyll concentration of 150 μg/ml. The quenching of QA fluorescence was induced by adding the cells sample to 2 ml reaction mixture after the background fluorescence became stable about 2 min after started the measurement.

## Additional Information

**How to cite this article**: Chen, X. *et al.* NdhV subunit regulates the activity of type-1 NAD(P)H dehydrogenase under high light conditions in cyanobacterium *Synechocystis* sp. PCC 6803. *Sci. Rep.* **6**, 28361; doi: 10.1038/srep28361 (2016).

## Supplementary Material

Supplementary Information
