# Supplementary material for: NdhV subunit regulates the activity of type-1 NAD(P)H dehydrogenase under high light conditions in cyanobacterium Synechocystis sp. PCC 6803
Source: Sci Rep. 2016 Jun 22;6:28361. doi: 10.1038/srep28361 (PMC4916593; doi:10.1038/srep28361)
Supplement: Supplementary Information [file srep28361-s1.pdf]

NdhV subunit regulates the activity of type-1 NAD(P)H dehydrogenase under high light conditions in cyanobacterium *Synechocystis* sp. PCC 6803

Xin Chen, Zhihui He, Min Xu, Lianwei Peng, Hualing Mi

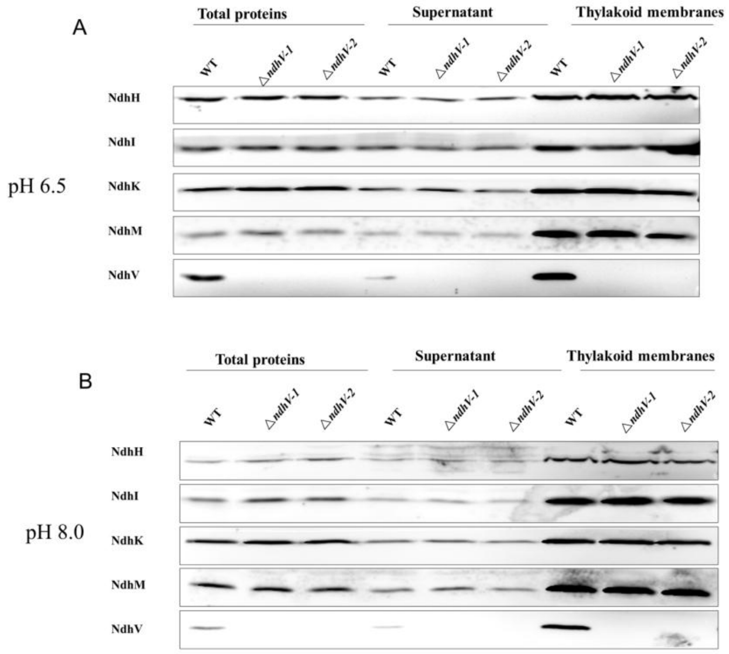

Figure 1S. Analysis of accumulation of Ndh subunits in WT and *ΔndhV* strains.
(A) Immunodetection of Ndh subunits in total proteins, supernatant and thylakoid membranes of WT and *ΔndhV* mutants in pH 6.5. Each sample was obtained from the cells cultured in pH 6.5. Immunoblotting was performed using antibodies against Ndh subunits (NdhH, NdhI, NdhK, NdhM and NdhV). Each lane was loaded with 30 μg proteins.
(B) Immunodetection of Ndh subunits in total proteins, supernatant and thylakoid membranes of WT and *ΔndhV* mutants pH 8.0. Each sample was obtained from the cells cultured in pH 8.0. Immunoblotting was performed using antibodies against Ndh subunits (NdhH, NdhI, NdhK, NdhM and NdhV). Each lane was loaded with 30μg proteins.

Supplemental Table 1

Primers List and Sequence

Primers used to construct the pMD-19T-Δ*ndhV* vector

Name	Sequence (5'-3')	Description
ndhV-up-F	GAGCCTGAGCCAATTGAGCCG	Amplification of upstream region
ndhV-up-R	ACAACGTGGCTTTCCCCACTGCGGGGGGTTTAGCTT	
ndhV-mid-F	CCGCAGTGGGGAAAGCCACGTTGTGTCTCA	Amplification of kanamycin resistance gene
ndhV-mid-R	GGGCTTCTCTTCGGGCGCTGAGGTCTGCCTCG	
ndhV-Dn-F	CTCAGCGCCCGAAGAGAAGCCCTTCACCGA	Amplification of downstream region
ndhV-Dn-R	TAGGTTTGATGGTGCTGCG	

Primers used to construct vectors to express protein to raise NdhV antibody.

Name	Sequence (5'-3')	Description
ndhV-F-KpnI	CGGGGTACCGGTGGCGCTCATTCCCTTTTACG	NdhV antibody
ndhV-R-BamHI	GCCGGATCCGGGTTGCCCCCTAGCCATTTTTGA	
